# Hierarchical Ordering of Reticular Networks

**DOI:** 10.1371/journal.pone.0036715

**Published:** 2012-06-06

**Authors:** Yuriy Mileyko, Herbert Edelsbrunner, Charles A. Price, Joshua S. Weitz

**Affiliations:** 1 Department of Mathematics, Duke University, Durham, North Carolina, United States of America; 2 Institute of Science and Technology Austria, Klosterneuburg, Austria; 3 Department of Computer Science, Duke University, Durham, North Carolina, United States of America; 4 Geomagic, Research Triangle Park, North Carolina, United States of America; 5 School of Plant Biology, The University of Western Australia, Crawley, Western Australia, Australia; 6 School of Biology and School of Physics, Georgia Institute of Technology, Atlanta, Georgia, United States of America; Université de Nantes, France

## Abstract

The structure of hierarchical networks in biological and physical systems has long been characterized using the Horton-Strahler ordering scheme. The scheme assigns an integer order to each edge in the network based on the topology of branching such that the order increases from distal parts of the network (e.g., mountain streams or capillaries) to the “root” of the network (e.g., the river outlet or the aorta). However, Horton-Strahler ordering cannot be applied to networks with loops because they they create a contradiction in the edge ordering in terms of which edge precedes another in the hierarchy. Here, we present a generalization of the Horton-Strahler order to weighted planar reticular networks, where weights are assumed to correlate with the importance of network edges, e.g., weights estimated from edge widths may correlate to flow capacity. Our method assigns hierarchical levels not only to edges of the network, but also to its loops, and classifies the edges into reticular edges, which are responsible for loop formation, and tree edges. In addition, we perform a detailed and rigorous theoretical analysis of the sensitivity of the hierarchical levels to weight perturbations. In doing so, we show that the ordering of the reticular edges is more robust to noise in weight estimation than is the ordering of the tree edges. We discuss applications of this generalized Horton-Strahler ordering to the study of leaf venation and other biological networks.

## Introduction

Networks and network theory have been utilized to represent and analyze the structure and function of a myriad of biological systems. These systems span scales from cells to ecosystems and include gene regulatory networks [1], [2], metabolic pathways [3], [4], disease dynamics [5], [6], food webs [7], [8], host-parasite webs [9], [10], and social interactions [11]–[13]. In the process, structural archetypes have been identified including scale-free behavior, motifs, modularity, the emergence of hubs, and small-world structure [11], [14]–[20]. However, these theories do not typically incorporate the spatial constraints that underlie the location and connections amongst nodes and edges. Indeed, there are many examples of delivery and distribution networks where nodes and edges are physical structures embedded in space, e.g., leaf venation networks [21], [22], cardiovascular networks [23], [24], cortical networks [25], root networks [26], ant trails [27] and road networks [28]. Hence, theory is also needed to characterize biological networks whose structure is strongly influenced by physical constraints (for a review, see [29]).

Although the theory of spatial networks is quite diverse, the theory as applied to resource delivery networks in biology often involves certain simplifying assumptions. For example, in fractal branching theory, a network is seen as a perfectly self-similar structure, e.g. a dividing binary tree [30]. A prominent theory of metabolic scaling in mammals assumes the cardiovascular system is a fractal whose physical dimensions have evolved to optimally transport fluid from the aorta to capillaries [31], [32]. An extension of this model to the above-ground structure of tree branches makes similar assumptions [33]. Both models have inspired a wide array of follow-up work with increased recognition that the original fractal branching assumption is overly simplistic [34]–[38]. For example, in reality, physical networks in biology have side branches and are not perfectly balanced binary trees [21]. Theories of side-branching resource delivery and distribution networks have their origins in the study of river networks. In a river network, streams merge together to form larger streams. However, small streams can merge into larger streams of all scales. The topological structure of river networks can be analyzed using the so-called Horton-Strahler order [39], [40]. This scheme assigns an integer number to every branch of the network. The numbers represent different levels of the branch hierarchy, with larger numbers corresponding to the larger stream segments in the network. The Horton-Strahler ordering is the basis for the characterization of the statistical properties of river networks [41], including the finding that river networks are fractal [42]. Moreover, the side-branching statistics first introduced by Tokunaga [43] can be used to characterize universal features of river networks and departures thereof [44].

Leaf venation networks are a prominent example of a physical delivery and distribution network whose structure possess numerous side branches. The structure of leaf venation networks has broad functional implications. For example, leaf vein density is positively correlated with photosynthetic rates [45] and also influences the extent to which leaves form a hydraulic bottleneck in whole plants [46], [47]. However, many leaves of higher plants (notably most leaves of angiosperm lineages), have reticulate venation networks, involving loops within loops [22]. It has been hypothesized that reticulate patterns allow leaves to maintain the supply of water and nutrients to and from photosynthetically active chloroplasts even when flow through some edges in the network is lost [48]–[51] due to mechanical damage or herbivory. Unfortunately, the Horton-Strahler ordering scheme developed for the analysis of river networks is not directly applicable to reticular networks. The reason is that loops lead to inconsistencies in the merging procedure in which a strictly hierarchical order is assigned to all edges.

In this paper we propose a method that generalizes the Horton-Strahler order to planar, weighted reticular networks. Such networks encompass a large class of physical networks, where weights can often be obtained by estimating dimensions of edges, such as branch widths, or other indicators of cost or importance. While coinciding with the Horton-Strahler order for branching networks, our method also assigns hierarchical levels to the loops of the network. Moreover, it categorizes the branches into the ones responsible for the formation of loops, and the ones forming the tree structure of the network. Edge weights play an important role in our algorithm, and we perform a theoretical analysis of possible effects of weight perturbations on the hierarchical levels. We find that the loop hierarchy is more robust to measurement error of network edge weights than is the tree hierarchy. In the past, comparisons of the statistical similarity between river networks and leaves have been proposed, albeit such comparisons are restricted to leaves without loops [52]. Hence, we also discuss applications of the current method to the characterization and comparison of reticulate leaf venation networks as well as obstacles to extending this method to a more general class of networks.

## Results

### A Graph Theoretic Approach to Horton-Strahler Ordering of Rooted Trees

**Figure 1:**
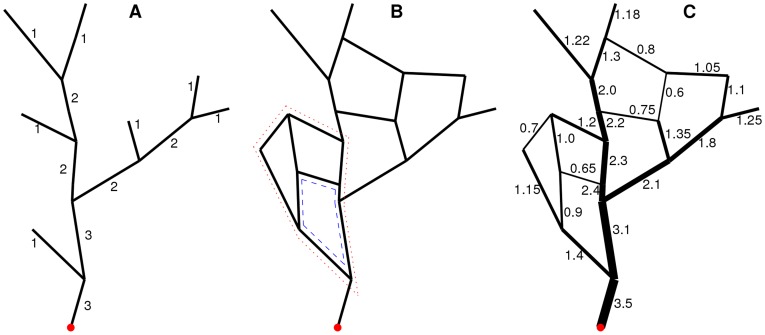
Examples of networks with hierarchical structure. A common “root” or outlet denoted by the red dot at the bottom of each network: (A) Horton-Strahler stream order of branch hierarchy in a tree network; (B) Reticular network with possible loop hierarchy: the, blue, dashed loop might be less important than the red, dotted loop; (C) Reticular network of (B) with weights.

We start by reviewing the algorithm for constructing the Horton-Strahler order. For the remainder of the paper, we shall adopt the language of graph theory [53], [54]. Note that in graph theory, the “leaves” of the network are those vertices which only have a single edge that connects to them. In this context, the input to the Horton-Strahler ordering algorithm is a rooted tree, 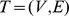, where *V* is the set of vertices and *E* is the set of edges. Given such a tree, the algorithm assigns a level, 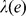, to each edge 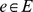 in the following way. First, assign level 1 to all edges connected to the leaves of *T*. Next, for each vertex having only one incident edge, *e*, with undefined 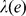, let *l* be the maximal level among the other incident edges. If there is a single incident edge of level *l*, then 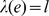. If there are two or more incident edges of level *l*, then 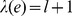. The result of this algorithm is illustrated in Fig. 1A. Conventionally in the study of river networks [42], this algorithm can be summarized by a single rule which states that the order of a downstream segment is equal to
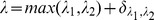
(1)where 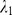 and 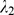 are the order of the two upstream segments that are merging and 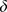 is the Kronecker delta.

It is clear, however, that if the network has loops, as in Fig. 1B, the algorithm cannot proceed because there always will be a vertex having more than one incident edge with an undefined level. Moreover, loops in this graph seem to also form a hierarchy. For example, the loop outlined in Fig. 1B by the red dotted line may belong to a higher level than the loop outlined by the blue dashed line. It turns out that such a hierarchy can be constructed and separated from the tree hierarchy if edges have weights and the graph itself is planar. An example of such a graph is shown in Fig. 1C, where the weights represent widths of the branches.

We developed an alternative, graph theoretic approach to implement the Horton-Strahler algorithm for the case when the tree *T* is binary and weighted (see Methods for the complete algorithm). Note that in a rooted tree, it is possible to partially order the edges such that 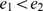 if there exists a path from the root to 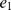 (e.g., an upstream edge) that passes through 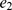 (e.g., a downstream edge). Further, we assume that there exist weights, 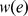, whose values are strictly increasing with respect to this order, that is, 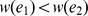 if 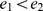. In the graph theoretic formulation of the Horton-Strahler algorithm we proceed as follows. First, we assign an order 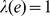 to all edges that connect to the leaves of the tree. Further, each edge is considered to be a disjoint component *c*. Next, we iterate through the remainder of the edges in order of increasing weight. For each edge *e*, we first evaluate whether the edge shares a vertex with a single component 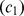 or with two components (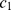 and 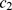). If an edge shares a vertex with a single component, then we merge 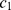 and *e* to form a new component whose order is the same as that of *e*. This merging represents the continuation of a subnetwork (e.g., the extension of a flowpath broken into segments). If an edge shares a vertex with two components, then the inclusion of edge *e* involves merging of two “upstream” components of the rooted tree (e.g., the merging of streams at a junction). The components are merged with the new edge to form a new (larger) component *c* which includes the edges in 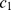, 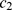 as well as the new edge *e*. The order 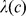 is assigned via the classic Horton-Strahler rule (see Eq. (1)) based on the order of the merged components, 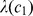 and 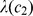. Further, the order of the assigned edge is set equal to that of the merged component, i.e., 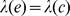.

### Ordering of Planar Weighted Graphs

We developed a graph theoretic procedure to generalize the Horton-Strahler order for planar graphs (see Methods for the complete algorithm). The input to this ordering procedure is a planar graph, 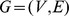 whose weights 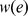 are assumed to be unique. In cases where weights are non-unique then ties will be resolved arbitrarily. This planar graph need not be a tree and may contain loops. The objective of this procedure is to order *both* the edges and the faces of the planar graph. In the previous Results section we showed how to merge disjoint components (i.e., 0-dimensional homology classes) to reproduce the Horton-Strahler ordering for rooted trees. Whereas for planar graphs, we are interested in constructing a hierarchy of loops which represent 1-dimensional homology classes. Hence, the basis for our graph theoretic procedure is to merge loops and to merge disjoint components. The key insights to our procedure stem from noting that (i) the boundary of a face of the graph *G* is a loop; (ii) we can merge two faces by removing a shared edge.

**Figure 2:**
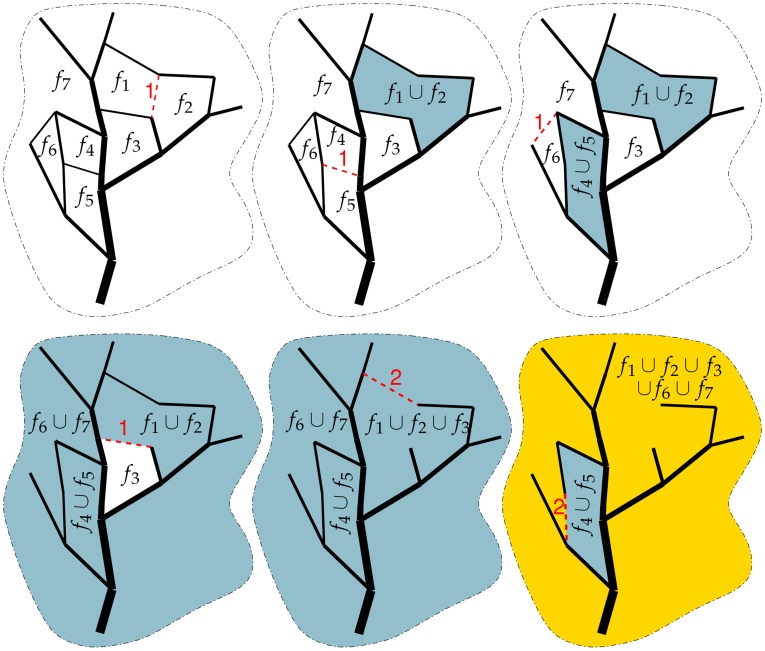
An illustration of the loop merging procedure. The merging is applied to the graph from Fig. 1C. Red, dashed edges are the ones removed during merging, the corresponding numbers show their levels. Levels of faces is encoded by the color: white faces have level 1, light blue faces have level 2, and gold faces have level 3. Note that 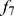 is the unbounded face.

The procedure to order planar weighted graphs can be summarized as follows. First, an order 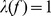 is assigned to all faces in the graph. We then iterate through edges in order of increasing weight. When a given edge is on the boundary of two distinct faces, then this edge is removed, creating a merged face. The order of this merged face follows the Horton-Strahler rule (see Eq. (1)) given the orders of the two faces. Similarly, the order of the edge to be removed is set equal to the minimum of the order of the two merged faces. A step-by-step illustration of loop merging applied to the tree in Fig. 1C is shown in Fig. 2. Notice that this procedure builds a rooted binary tree, where leaves correspond to the faces of *G*, and the rest of the vertices correspond to unions of these faces. The assignment of levels in this tree follows the original Horton-Strahler algorithm. It is also useful to remember that faces of *G* are vertices of its dual graph, 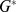, and merging faces of *G* can be thought of as adding an edge to 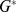. Hence, the two merging procedures that we described are, in some sense, dual. We shall refer to the binary tree of faces as the *co-tree* of *G*, and denote it by 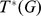.

**Figure 3:**
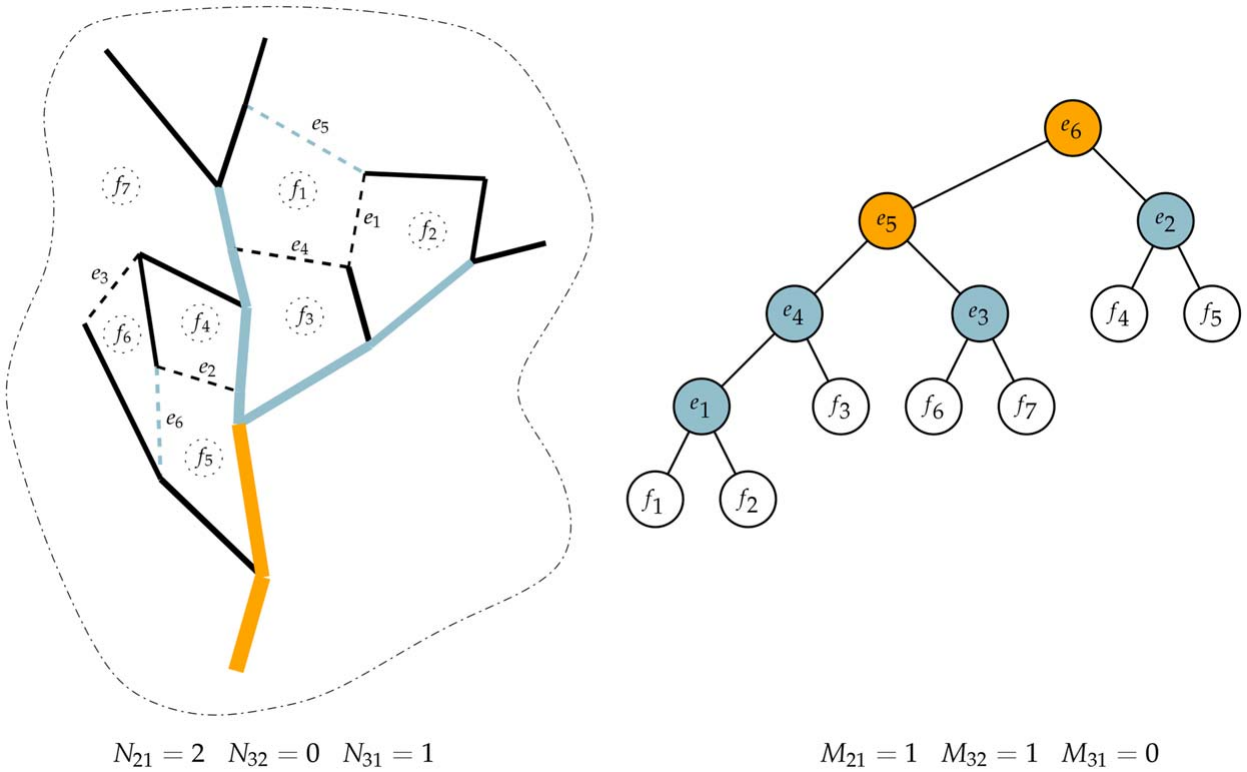
Example of hierarchical levels. The levels are assigned to the loops and branches of the network from Fig. 1C. Edge levels are shown on the left, where black edges have order 1, light blue edges have order 2 and gold edges have order 3; reticular edges are dashed. Face levels are shown in the co-tree on the right, where white nodes have order 1, light blue nodes have order 2 and gold nodes have order 3. Leaves of the co-tree are labeled by the corresponding faces while other nodes are labeled by the reticular edges causing the merger of the two child nodes. Numbers 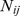 are Tokunaga statistics for the spanning tree and indicate the number of edges of level *j* joining with edges of level *i* [43]. Similarly, 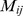 are Tokunaga statistics for the reticulate co-tree and indicate the number of edges and faces of level *j* merging with edges of level *i*. For both *M* and *N*, statistics are only collected when 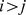.

The construction of 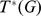 removes edges from *G* which are responsible for the existence of loops. We shall call such edges *reticular*. Assignment of levels for such edges is based on the assumption that a merger should not be more significant than any of the merging elements. Notice that after removing reticular edges from *G* we have a spanning tree of *G*, which we denote by 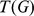. This tree captures the tree-like structure of the original network, and we can assign hierarchical levels to its edges using the original Horton-Strahler algorithm. We only need to determine which vertex should be the root, and we do this by finding the vertex with a single incident weight of maximum weight. Hence, as noted in the Methods, the final step is to aply the Horton-Strahler ordering to the remainder of the graph (which is a rooted tree). The result of the complete algorithm applied to the tree in Fig. 1C is provided in Fig. 3.

The algorithm produces three types of output. First, it provides a unique set of orders to those edges involved in the non-reticulate component of the network (Figure 3 - left panel). Second, it provides a unique set of orders to those edges involved in the formation of loops (Figure 3 - right panel). Further, one can also calculate the side-branching statistics associated with both orderings. The side-branching statistics, i.e., “Tokunaga” statistics [43], for a conventional non-loopy tree are summarized by the numbers 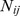 which are the number of edges of level *j* that join with edges of level *i*. Because of the ordering process, these statistics are evaluated for 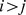. These numbers can also be divided by the number of absorbing edges, i.e., the total number of edges of level *i* to yield an average number of side-branches per segment. Here, the algorithm produces two sets of Tokunaga statistics, the numbers 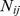 for the side-branching of tree edges (Figure 3 - left panel) and 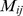 for the side-branching of reticulate edges (Figure 3 - right panel).

### Sensitivity of Planar Network Ordering to Weight Perturbations

Clearly, edge weights play an important role in the construction of both loop and tree hierarchies. Unfortunately, weight estimation done in practice is often imprecise, so the order in which the algorithm iterates through the edges may be perturbed. In this section we investigate how such a perturbation affects the loop and tree hierarchies.

**Figure 4:**
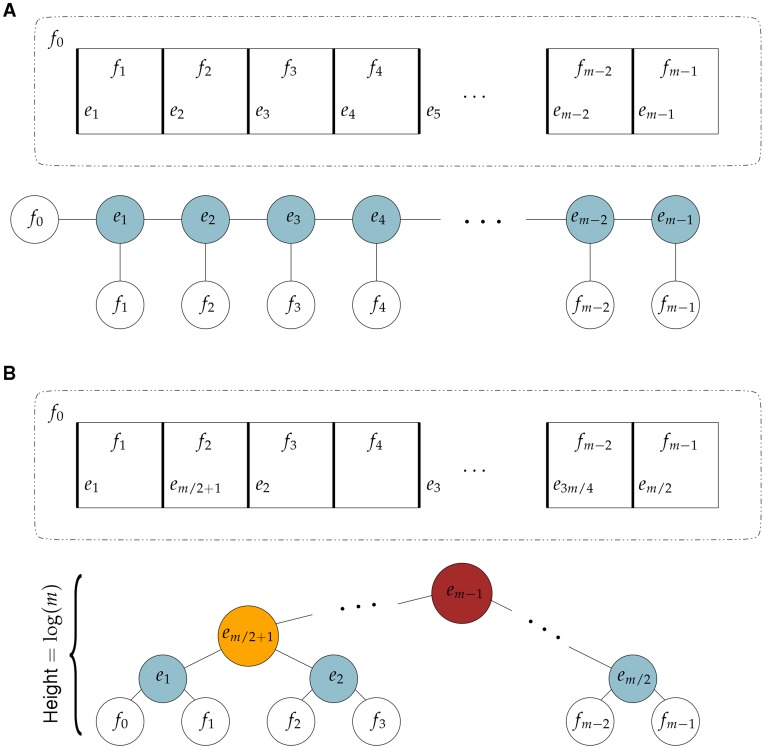
Example of the two extreme cases of the loop hierarchy. The network has *m* faces, where 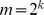 for some integer 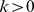. 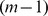 of these faces are adjacent squares and the other one is the unbounded face. Vertical edges are removed before horizontal edges as follows: (A) The edges are removed sequentially from left to right. The corresponding co-tree has the shape of a “comb” and the maximal hierarchical level is 2; (B) The edges are removed from left to right skipping every second edge. The process is repeated until all vertical edges except the rightmost one are removed. The corresponding co-tree has the height 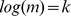 which is the maximal hierarchical level.

We start by considering the worst possible change in the hierarchical levels of loops. Notice that the highest level in the hierarchy of loops can be as low as 2. This happens when the first reticular edge creates a level 2 face and every other reticular edge merges a level 1 face with the only level 2 face (see Fig. 4A). On the other hand, the highest level in the loop hierarchy can be as high as 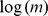, where *m* is the number of faces. This happens when level 1 faces are merged only with level 1 faces until only faces of level 2 are left, then level 2 faces are merged with level 2 faces until only faces of level 3 are left, and so on (see Fig. 4B). It is clear from the example in Fig. 4 that there is a permutation of edges that can change the loop hierarchy from one of the extreme cases to the other. However, in practice such a permutation would generally result in from a significant perturbation in weights. For small perturbations, it is more likely that only a few transpositions of edges will occur.

Let 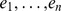 be the order of edges with respect to their weights. We shall now analyze how the structure of 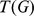 and 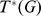 changes when a single transposition occurs, that is, when the order of 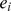 and 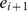 is swapped. First, we notice that there will be no changes to the structure of the co-tree or the spanning tree if 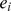 and 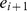 are both tree edges, or if 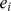 is a tree edge and 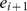 is a reticular edge. Hence, there are two cases to consider: when both 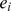 and 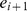 are reticular, and when 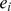 is a reticular edge and 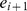 is a tree edge. In the former case, we can regard reticular edges as edges of the co-tree. We see then that swapping the two edges may shift a subtree of the co-tree only one level up or down. Therefore, it is reasonable to expect that hierarchical levels of loops will change at most by one. The case of a reticular edge and a tree edge is more complicated. Such a transposition may lead to detaching a subtree of the remaining spanning tree and attaching it at a different place. This may have a drastic effect on the tree hierarchy. A detailed analysis of the two cases justifying the above conclusions is present below.

#### Case 1


**Figure 5:**
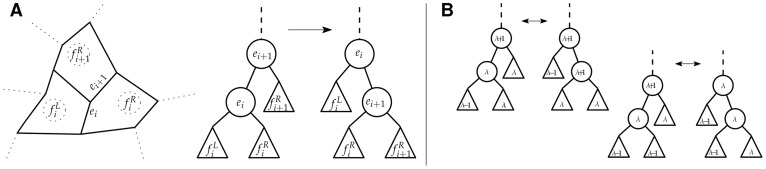
Effect of a single transposition of two reticular edges. (A) the part of the network containing the two edges being transposed and the effect of the transposition on the structure of the co-tree; (B) possible level changes caused by the transposition.

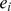
 and 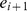 are both reticular. Only the co-tree can be affected in this case. Let 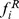, 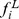 and 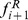, 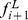 be the faces merged by removing 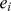 and 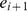, respectively. Also, let 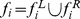 and 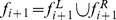. Notice that if 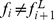 and 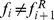, then 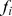 is not a child of 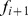 in 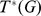), and there will be no changes to the structure of the co-tree. Suppose that 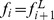 (the case when 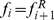 follows the same argument). Then 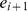 is adjacent to either 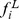 or 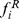; let us assume it’s 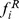. Removing 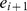 before 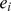 leads to merging 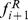 with 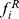 first, and then merging the resulting face with 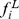. The corresponding change in the tree structure, shown in Fig. 5, is a single rotation around 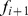. Possible changes in the levels of the nodes involved in the rotation are also shown in Fig. 5. We can see that these levels can change at most by one. However, in the worst case the change in levels may propagate up 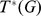 all the way to the root.

#### Case 2


**Figure 6:**
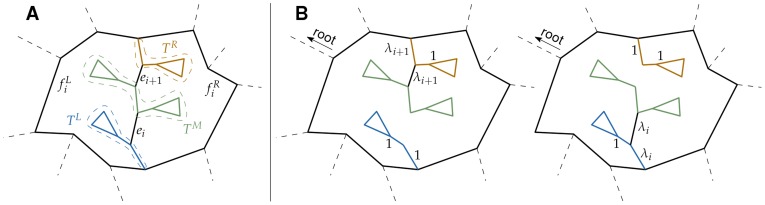
Effect of a single transposition of a reticular edge and a tree edge. (A) The part of the network containing the two edges being transposed. The brown, blue, and green triangles (and edges) denote the subtrees adjacent to the edges. (B) The effect of the transposition on the structure of the spanning tree and its hierarchical levels.

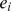
is a reticular edge and 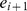 is a tree edge. Let 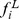 and 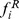 be the two faces merged by removing 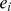. Notice that there will be no changes in the structure of 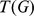 or 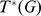 if 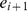 is not adjacent to both 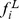 and 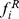. So, let 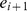 be adjacent to 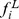 and 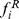. Then removing 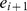 before 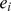 merges the same 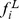 and 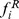, so no changes to the structure of the co-tree happen. However, 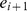 turns into a reticular edges, and 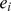 becomes a tree edge. Consequently, the structure of the spanning tree changes. Let 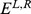 be the set of edges incident to both 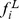 and 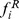, and let 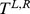 be the tree formed by the edges in 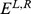 and the edges connected to 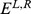 and having only 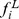 or 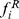 as an adjacent face (see Fig. 6). Removing 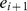 and 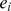 splits 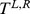 into three trees, 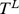, 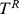, and 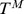, such that 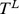 and 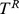 are connected to the boundary of 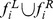, and 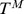 is not (Fig. 6). If 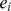 is removed before 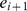, then 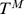 is connected to 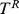 by 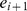. However, if the transposition happens and 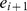 is removed before 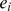, then 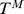 is connected to 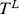 by 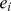 (Fig. 6). To understand the effect of such a change on hierarchical levels, we first assume that 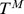 does not contain the root of 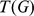. Let 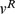 be the vertex incident to 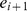 and 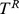, and let 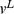 be the vertex incident to 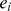 and 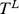. Also, let 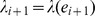, where 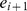 is regarded as an edge in 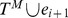 rooted at 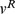, and let 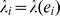, where 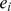 is regarded as an edge in 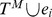 rooted at 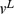. Denote by 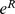 the edge of 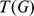 which is next to 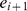 in the path from 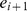 to the root of 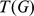, and by 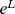 the edge of 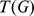 which is next to 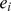 in the path from 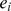 to the root of 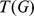. Then we can see that removing 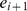 before 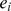 can decrease the level of 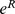 by at most 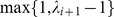. At the same time, the level of 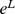 can increase by at most 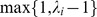. In the worst case, these changes can propagate up 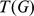 all the way to the root. The case when the root of 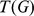 belongs to 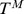 can lead to more drastic changes. In this case, removing 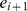 before 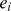 leads to recomputing levels of all edges in 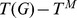 by changing the root from 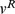 to 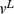. Again, this change can then propagate further to the root of 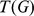.

## Discussion

We have shown that the hierarchy of loops often observed in reticular physical networks can be defined explicitly using a generalization of the Horton-Strahler order. To obtain such a generalization we regard the network as a weighted graph, with weights corresponding to the widths of the network branches. Noticing that the Horton-Strahler order can be computed by analyzing how specific disjoint components (sub-networks) of a (non-reticular) network are merged as the edges are *added* in the order of increasing weight, we show that the hierarchical order of loops in a weighted planar graph can then be computed by analyzing how the *faces* of the graph are merged as we *remove* the edges in the order of increasing weight. This approach naturally classifies graph edges into reticular edges, which are responsible for loop formation, and tree edges, which constitute a spanning tree of the graph. Hence, both the loop and the tree hierarchies can be computed.

Being able to compute hierarchical levels for loops creates new possibilities for analyzing the structure of reticular networks. By means of analogy, river networks can be compared by representing their connectivity in terms of side-branching statistics [43]. These statistics depict the ways in which smaller streams connect to larger streams at all scales of the network [44]. A similar procedure could be applied to leaf networks. For example, the current algorithm decomposes reticulate networks into a binary-tree for the loop hierarchy and a separate binary tree for the tree hierarchy. Both networks have associated Horton-Strahler orders and therefore their structure can be estimated using Tokunaga statistics. Recent innovations in software now permit semi-automated extraction of the dimension and connectivity of entire leaf vein networks and the areoles that veins surround [55]. Hence, greater quantification of leaf vein networks from across a wide range of biological diversity will soon be available for which to analyze leaf development, variation across environmental gradients and in paleobotanical studies. Current attempts to compare reticulate structure have largely focused on the density of areoles (i.e., network faces) as a proxy for the “loopiness” of the network [56]. The current study will provide additional metrics to compare the detailed branching structure of reticulate networks.

An important caveat to keep in mind when comparing reticulate network structure is that estimating weights in physical networks is by no means a trivial problem. Therefore, we have performed a theoretical analysis of possible changes in the loop and tree hierarchies due to perturbations in edge weights. We have shown that the worst possible change in the loop hierarchy is attainable, but requires a significant perturbation of weights. Taking into account that small perturbations are likely to cause only a few transpositions in the order in which the edges are removed, we have shown that a single such transposition can change the hierarchical levels of loops at most by one. We have also shown that the change in the hierarchical levels of the remaining spanning tree can be arbitrarily large even when a single transposition is performed. It is important to note that in either case the change does not happen for every transposition. Rather, the transposed edges have to satisfy a particular condition, which may happen rarely in practice. The latter claim is supported by the numerous successful applications of the Horton-Strahler order. While the method itself does not depend on any weights, the connectivity of the network is obtained by analyzing digital elevation map which contain noise [57], [58]. In particular, the difference between the correct and the computed connectivity may be exactly the same as the difference in the connectivity of our spanning tree caused by transposing two edges. Hence, the resulting hierarchy may be drastically different from the correct one. Nevertheless, the Horton-Strahler order has been successfully used for over five decades despite the potential instability identified here [39]–[42], [57]. We suggest that empirical characterizations of reticulate planar networks include randomization analysis on edge weights to identify the robustness of claims regarding statistical structure of side-branching of the tree and co-tree.

Many biological and physical systems are represented by non-planar physical networks [29], [59] and computing hierarchical levels of loops in such networks is still an open question. While our method can be applied to obtain the tree hierarchy of such networks, the loop hierarchy cannot be computed in this case because the algorithm relies on the fact that any loop in a planar network corresponds to a union of faces. In the mathematical language, (boundaries of) faces of a planar graph form a canonical basis for loops (1-dimensional homology classes). Such a canonical basis is not present in non-planar graphs. It is not clear at this point how to handle the non-planar case. Perhaps a method for computing loop hierarchies which is not based on the widths of the network branches could provide an answer. We hope that our approach of using algebraic topology language to deal with nodes and loops of networks will prove useful in developing such a method and complement other approaches.

## Methods

### Algorithm for Graph Theoretic Ordering of Rooted Trees

Here we present a graph theoretic algorithm for ordering the edges within a rooted tree, 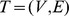, where *V* is the set of vertices and *E* is the set of edges. Consider the case when the tree *T* is binary and weighted. Let 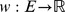 be the weight function, that is, 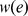 is the weight of an edge 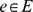. Since the tree is rooted, there is a partial order defined on *E* as follows: 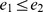 if there is a path from the root to 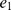 which passes through 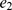 (in other words, 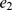 is closer to the root than 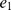). Let us assume that the weight function is strictly increasing with respect to this order, that is, 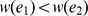 if 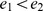. In this case, the Horton-Strahler order can be computed using the following procedure:

- Let 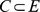 be the set of edges incident to leaves of T, regarded as a set of disjoint components. For each 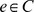 let 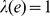.
- Iterate through (the rest of the) edges in order of increasing weight. For each edge e do the following:
  1. If e shares a vertex with a single component 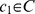, then merge 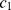 and e into a new component c, and let 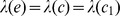.
  2. If e shares a vertex with two components 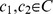, then merge 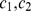, and *e* into a new component *c*, and assign levels as follows:
    * If 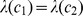, then 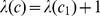.
    * If 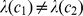, then 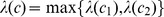.
    * 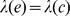.

### Algorithm for Graph Theoretic Ordering of Planar Weighted Graphs

Here we present the algorithm for constructing the generalized Horton-Strahler order of a weighted planar graph. Let 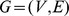 be a planar graph, not necessarily a tree, and again let 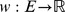 be a weight function. We shall assume that *w* is injective (i.e., all weights are unique). Otherwise, the ties will be resolved arbitrarily. The merging procedure for computing the Horton-Strahler order works with disjoint components, which, in the language of algebraic topology, are 0-dimensional homology classes. Loops, on the other hand, are 1-dimensional homology classes. Hence, we may try to construct a hierarchy by merging loops. Notice that the boundary of a face of the graph *G* is a loop, and we can merge two neighboring faces by removing a shared edge. Using these two observations, we obtain the following merging procedure for loops:

- Sort the edges so that 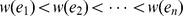, where 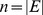 is the number of edges.
- Let 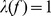 for each face f.
- Iterate through 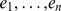 and do the following:
  1. If 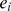 is adjacent to a single face, skip to the next edge.
  2. If 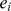 is adjacent to two distinct faces 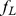 and 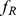, remove 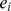 from the graph, let 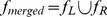, and assign the levels as follows:
    * If 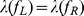 then 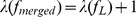.
    * If 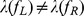 then 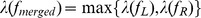.
    * 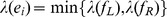.

This algorithm will remove reticular edges from *G*, generating a spanning tree of *G*, which we denote by 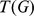. Hence, we augment the procedure for constructing the loop hierarchy by the following statement:

- Apply the Horton-Strahler ordering to the remainder of the graph, 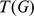, (which is a rooted tree).
